# Metabolic Specialization and Codon Preference of Lignocellulolytic Genes in the White Rot Basidiomycete *Ceriporiopsis subvermispora*

**DOI:** 10.3390/genes11101227

**Published:** 2020-10-20

**Authors:** Alex Gonzalez, Gino Corsini, Sergio Lobos, Daniela Seelenfreund, Mario Tello

**Affiliations:** 1 Laboratorio de Microbiología Ambiental y Extremófilos, Departamento de Ciencias Biológicas y Biodiversidad, Universidad de los Lagos, Osorno 5290000, Chile; alex.gonzalez@ulagos.cl; 2 Instituto de Ciencias Biomédicas, Facultad de Ciencias de la Salud, Universidad Autónoma de Chile, Santiago 8910132, Chile; gino.corsini@uautonoma.cl; 3 Departamento de Bioquímica y Biología Molecular, Facultad de Ciencias Químicas y Farmacéuticas, Universidad de Chile, Santiago 8380492, Chile; slobos@uchile.cl (S.L.); dseelen@ciq.uchile.cl (D.S.); 4 Laboratorio de Metagenómica Bacteriana, Centro de Biotecnología Acuícola, Facultad de Química y Biología, Universidad de Santiago de Chile, Santiago 9170002, Chile

**Keywords:** Codon bias, *Ceriporiopsis subvermispora*, lignocellulose degrading system

## Abstract

*Ceriporiopsis subvermispora* is a white-rot fungus with a high specificity towards lignin mineralization when colonizing dead wood or lignocellulosic compounds. Its lignocellulose degrading system is formed by cellulose hydrolytic enzymes, manganese peroxidases, and laccases that catalyze the efficient depolymerization and mineralization of lignocellulose. To determine if this metabolic specialization has modified codon usage of the lignocellulolytic system, improving its adaptation to the fungal translational machine, we analyzed the adaptation to host codon usage (CAI), tRNA pool (tAI, and AAtAI), codon pair bias (CPB), and the number of effective codons (Nc). These indexes were correlated with gene expression of *C. subvermispora*, in the presence of glucose and Aspen wood. General gene expression was not correlated with the index values. However, in media containing Aspen wood, the induction of expression of lignocellulose-degrading genes, showed significantly (*p* < 0.001) higher values of CAI, AAtAI, CPB, tAI, and lower values of Nc than non-induced genes. Cellulose-binding proteins and manganese peroxidases presented the highest adaptation values. We also identified an expansion of genes encoding glycine and glutamic acid tRNAs. Our results suggest that the metabolic specialization to use wood as the sole carbon source has introduced a bias in the codon usage of genes involved in lignocellulose degradation. This bias reduces codon diversity and increases codon usage adaptation to the tRNA pool available in *C. subvermispora*. To our knowledge, this is the first study showing that codon usage is modified to improve the translation efficiency of a group of genes involved in a particular metabolic process.

## 1. Introduction

The main carbon source synthesized through photosynthesis which plays a central role in the carbon cycle of the planet is lignocellulose. Lignin, one of the compounds of lignocellulose, is a recalcitrant, aromatic, and amorphous polymer that protects lignocellulose from microbial attack. A small group of filamentous fungi from the basidiomycete phylum is unique in its ability to efficiently degrade lignocellulose [1]. Together they are collectively known as white rot fungi, developing an enzymatic machinery that allows degradation of the three main components of lignocellulose: lignin, cellulose, and hemicellulose. White-rot fungi mineralize lignin as a strategy to access cellulose and hemicellulose, whose sugar moieties are used as carbon and energy sources. Degradation of these aromatic and carbohydrate polymers progresses by different mechanisms. While mineralization of lignin is carried out by free radicals generated enzymatically [2,3], the degradation of cellulose and hemicellulose into its constituent sugars occurs through the combination of direct enzymatic hydrolysis and partial hydrolysis by enzymatically generated free radicals [4].

*Ceriporiopsis subvermispora* is a lignocellulose degrading white-rot basidiomycete that mineralizes lignin using a machinery composed of manganese peroxidase, versatile peroxidase, lignin peroxidase, and laccase, as well as an accessory system generating lipoperoxides [5,6,7,8,9]. Biochemical and genetic evidence shows that manganese peroxidases and laccases are encoded by a family of structurally related genes [6,8,10,11,12]. Hydrolysis of cellulose and hemicellulose is also achieved by a suite of cellulases and hemicellulases, which are encoded by multigene families [8]. Gene expression of enzymes involved in lignocellulose digestion and mineralization is induced by compounds present in wood, as shown by research using Northern blot, RT-PCR, and microarrays [8,13,14,15,16,17]. These studies also indicate that expression levels differ among members of a given multigene family [8,11,18]. Although selective pressure imposed by lignocellulose metabolism is reflected by an increase in the copy number of lignocellulolytic genes in *C. subvermispora* and other white-rot fungi [8], it is unknown whether this condition has influenced the composition of synonymous codons in these genes.

Preference for use of some synonymous codons (codon bias) is influenced by two mechanisms, mutational bias, and translational selection. Mutational bias establishes that the codon preference of a gene is determined by the GC content of the organism or the region where the gene is located in the genome [19]. On the other hand, translational selection establishes that codon bias is determined by the influence of synonymous mutations on the translational process [20,21]. Highly expressed genes, such as those coding for ribosomal proteins, tend to have a greater bias in the use of synonymous codons, preferentially using codons which are most represented in the genome [22]. In model organisms such as *Escherichia coli* and *Saccharomyces cerevisiae*, tRNAs that decode frequently used codons tend to have a larger gene copy number [23,24]. Thus, genes with frequently used codons are more adapted to the tRNA pool and consequently have more tRNAs available for their translation, which in turn improves the efficiency of protein synthesis. 

In this work, we compared codon usage, adaptation to the tRNA pool, and codon bias of *C. subvermispora* genes involved in lignocellulose metabolism and genes related to other cell processes. Our results show that selective pressure imposed by the use of lignocellulose has specifically modified codon usage of genes involved in the utilization of lignocellulose, favoring an increase in translational efficiency with respect to genes not involved in this process.

## 2. Materials and Methods 

### 2.1. Sequences

Genome sequences from *C. subvermispora* were downloaded from https://mycocosm.jgi.doe.gov/Cersu1/Cersu1.home.html.

### 2.2. Microarray Data 

Microarray data were downloaded from the Gene Expression Omnibus Database, accession no. GSE34636 [8].

### 2.3. Determination of the Gene Copy Number Coding for tRNAs 

Genes encoding tRNAs were identified using the tRNAscan-SE program, using unmasked scaffolds assembled from the genome sequence of *C. subvermispora* [8,25].

### 2.4. Determination of Codons and Codon Pair Frequencies 

The frequency of codon usage and codon pairs was determined from the coding sequences of *C. subvermispora*. To calculate the frequency of codon usage the program JEMBOSS through the CUSP routine was used (http://emboss.sourceforge.net/Jemboss/) [26]. A written script in Python language was used for determining the frequency of codon pairs [27].

### 2.5. Determination of Bias in Codon-Pair Usage

Bias in codon-pair usage was determined as described by Coleman [28]. The observed frequency of amino acid pairs was deduced based on the sum of all the codon pairs that codify for the same combination of amino acids. Subsequently, we used these frequencies to calculate the CPS index (codon-pair score) using the equation described by Mueller et al. 2006 [29] (Equation (1)), where *f*(*AB*) corresponds to the frequency of the appearance of the AB codon pair expressed in parts-per-thousands with respect to the total number of codon pairs in the genome. *f*(*A*) and *f*(*B*) correspond to the abundance of each of the individual codons expressed in parts-per-thousands with respect to the total number of codons in the genome. *f*(*X*) and *f*(*Y*) correspond to the amino acids codified by the A and B codons, respectively, and *f*(*XY*) is the abundance of the amino acid pairs in all the proteins of the organism under study. The CPS values of the 3271 codon pairs in *C. subvermispora* can be found in the Supplementary Material (Table S1). We calculated the bias in codon usage as the arithmetic mean of the codon-pair score of a gene (CPS) as described by Coleman et al. (2006) (Equation (2)), in which *i* is the *i*th codon pair of a gene and 1 is the total length of the gene expressed in the quantity of codified amino acids.

(1) $$CPS=Ln\;\left(\frac{f\left(AB\right)}{f\left(A\right)\times f\left(B\right)}\times \frac{f\left(X\right)\times f\left(Y\right)}{f\left(XY\right)}\right)$$

(2) $$CPB=\frac{\sum_{i}^{l}CPS_{i}}{l-1}$$

### 2.6. Calculation of Relative Synonymous Codon Usage (RSCU) and Codon Adaptation Index (CAI)

The Relative Synonymous Codon Usage (RSCU) and Codon Adaptation Index (CAI) were calculated with the Emboss program [30] based on the frequency of codon usage of *C. subvermispora*.

### 2.7. Determination of Adaptation to the tRNA Pool 

The adaptation to the tRNA pool of genes present in *C. subvermispora* was determined by calculating the values of tAI and AAtAI [31]. We calculated tAI as established by dos Reis [24] (Equations (3) and (4)), estimating the relative abundance of tRNAs from the number of copies of each tRNA gene in the *C. subvermispora* genome. The number of copies of each tRNA gene was determined with the tRNAscan-SE program [25] in Linux, which was used to analyze the unmasked assembled *C. subvermispora* genome.

(3) $$W_{i}=\sum_{j=1}^{n_{i}}\left(1-S_{ij}\right)tGNC_{ij}$$

*W*_i_ is the relative adaptiveness of the *i*th codon to the tRNA pool, *n*_i_ is the number of tRNA isoacceptors that recognize the *i*th codon, tGCN_ij_ is the number of copies of the *j*th tRNA gene that recognizes the *i*th codon, and *S*_ij_ is the selective constraint in the efficiency of codon-anticodon pairing.

(4) $$tAI_{g}={\left(\prod_{k=1}^{l_{g}}w_{ikg}\right)}^{1/l_{g}}$$

The adaptation of a gene to the tRNA pool is calculated according to Equation (4), in which w_i_ is defined as the ratio between *W*_i_ and *W*_max_ (*W*_i_/*W*_max_), ikg is the codon defined by the *k*th triplet of gene g, and l_g_ is the length of gene g in codifying codons.

### 2.8. Determination of AAtAI 

Briefly, AAtAI was calculated using Equation (5).

(5) AAtAIg=(∏k=1lgwikgAA)1/lg

^AA^*w*_i_ is defined as the ratio between *W*_i_ and W_AAmax_ (*W*_i_/*W*_AAmax_), where *W*_AAmax_ is the highest value of Wi among codons coding for the same amino acid. *i*kg is the codon defined by the *k*th triplet of gene *g*, and *l*_g_ is the length of gene g in codifying codons. As AAtAI is calculated from Equation (5), which is similar to the calculation of CAI, we used the EMBOSS software [30], entering *W*i data in replacement of the frequency of codon usage. Wi was calculated using the procedure described for tAI in Equation (3).

### 2.9. Determination of effective Number of Codons (Nc)

The number of effective codons for the *C. subvermispora* genes was calculated using the CodonW program (http://codonw.sourceforge.net/).

### 2.10. Phylogenetic Analysis

Multialignment between sequences of tRNA genes was performed using ClustalW [32]. The parameters were set up to align sequences using IUB as substitution matrix. The evolutionary history was inferred using the Neighbor-Joining method [33]. Interior branch test with a bootstrap of 1000 was used to analyze confidence of the tree [34]. The evolutionary distances were computed using the Maximum Composite Likelihood method [35]. The rate of variation among sites was modeled with a γ distribution (shape parameter = 1). Multialignment and evolutionary analyses were conducted in MEGA5 [36].

### 2.11. Graphs and Statistical Methods 

The program SigmaPlot 11 was used for graphs and statistical tests. The significance of the differences or correlations among the data groups obtained was evaluated with the Rank Sum Test non-parametric test for comparing two groups and the non-parametric Spearman Rank Order test for correlations, using a *p*-value < 0.05 as a cutoff.

## 3. Results

### 3.1. C. subvermispora tRNAs 

**Figure 1:**
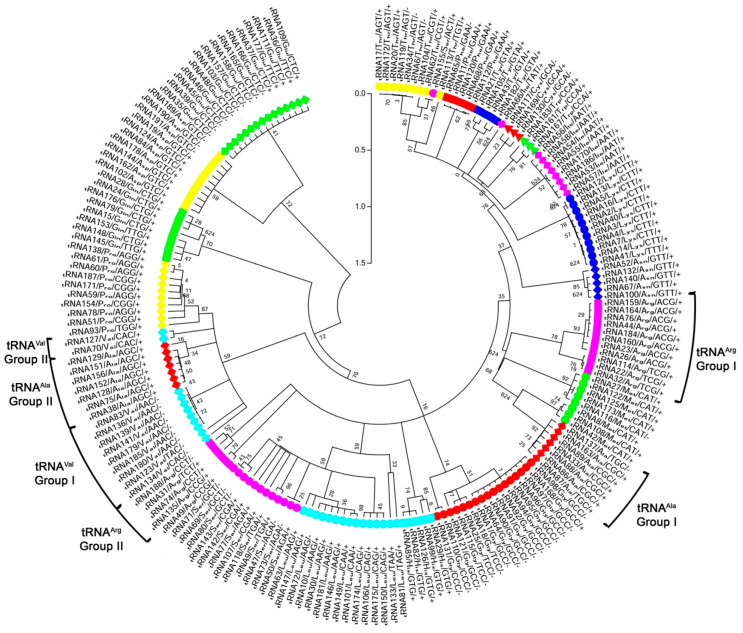
Phylogenetic analysis of tRNAs from *Ceriporiopsis subvermispora*. tRNAs were numbered according the tRNAScan-SE output. Prediction of amino acid charge, sequence of anticodon, and presence (+) or absence (−) of introns are indicated. Each tRNA type is indicated with a different symbol and color.

Genome analysis of *C. subvermispora* by tRNAScan-SE identified a total of 192 tRNAs in 32 scaffolds (Figure 1). About 72% of the tRNA genes presented introns (Table 1). The scaffold with the highest number of tRNA genes was scaffold 1, which contains 20 copies of various tRNA genes (Table S2). The tRNA with the highest number of gene copies corresponded to tRNAs charging glycine, with 17 gene copies distributed in eight scaffolds (scaffolds 1, 2, 5, 7, 9, 10, 13, and 20). The tRNAs for cysteine and tryptophan presented the lowest number of gene copies, each with three gene copies in three scaffolds (Table 1).

### 3.2. Phylogenetic Analysis of C. subvermispora tRNA Genes 

To determine whether the high number of tRNA genes charging the same amino acid corresponds to related gene copies, a phylogenetic reconstruction and evolutionary distance calculation were performed using the tRNA sequences identified by the tRNAscan-SE program. Phylogenetic reconstruction indicated that most tRNA genes that code for the same amino acid group together, with the exception of the tRNA genes that charge arginine, valine, and alanine (Figure 1), which form two groups in each case. For tRNAs loading arginine, Group I comprises genes presenting anticodons with the WCG sequence. In contrast, group II presents a YCK anticodon consensus sequence. In tRNAs that load valine, Group I corresponds to tRNA genes without introns, while group II includes all valine tRNA genes containing introns. Genes coding for group I of alanine tRNAs exhibit anticodons with the consensus sequence YGC, whereas, the anticodon sequence is AGC for group II (Figure 1). The tRNA genes 66 and 155 also show a different pattern. tRNAScan prediction indicates that the amino acid loaded by tRNA155 should be serine, however, the sequence of this gene grouped with threonine charging tRNA genes. Additionally, the tRNA66 gene is expected to load isoleucine, though this gene does not group with isoleucine charging tRNAs (Figure 1). To identify tRNA genes that are repeated, the evolutionary distance between different tRNA genes was calculated. Genes with values of evolutionary distance equal to 0.000 were selected. This analysis identified 15 tRNA genes that are repeated between two and ten times. The group of tRNA genes that showed the greatest expansion corresponds to those tRNAs carrying glutamic acid and glycine. One glycine tRNA gene is repeated twice and the other is repeated ten times. The tRNA gene for glutamic acid is also repeated ten times (Table S3). These genes are scattered along the genome, with the exception of tRNA genes 91, 92, 94, 95, 96, and 97, which code for glycine and are located at adjacent positions.

### 3.3. tRNA Abundance and Codon Usage in C. subvermispora

The expansion of certain families of tRNAs in the genome of *C. subvermispora* could be the result of an evolutionary pressure to increase their expression. In some organisms such as *E. coli* and yeast, the number of copies of a tRNA gene is proportional to the abundance in the genome of the decoded codon [24]. This proportionality is explained because during translation process, aminoacyl-tRNAs that decode frequently used codons have a higher rate of consumption. To sustain adequate translation, cells must balance synthesis and consumption rates of aminoacyl-tRNAs. The increase in copy number of a gene that encodes a tRNA that recognizes a frequently used codon, allows increasing expression of this tRNA and its aminoacylated form to balance its rate of consumption.

**Figure 2:**
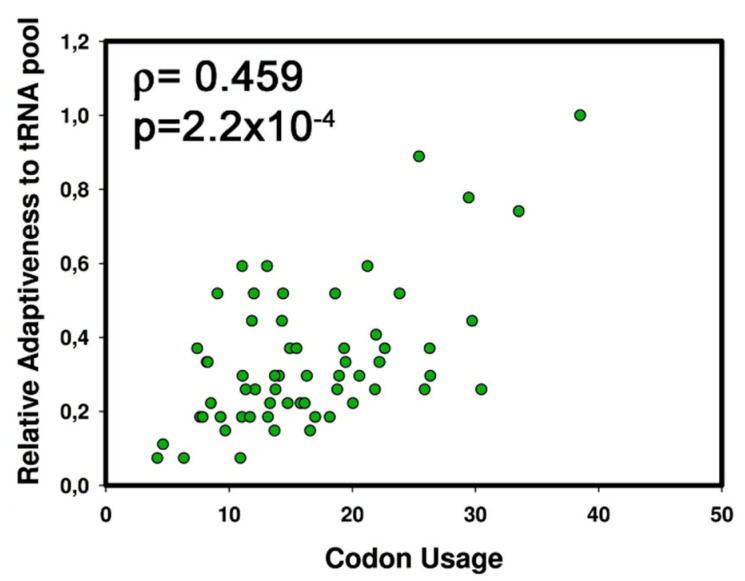
Correlation between codon usage and relative adaptiveness to the tRNA pool in *C. subvermispora*. Correlation between the relative adaptiveness to the tRNA pool and codon frequency usage (per thousand) of the 61 codons is shown.

To test if the expansion of certain tRNA families in *C. subvermispora* is related to the increment of the specific codons, we assessed whether there is a correlation between the frequency of codon usage and the amount of tRNA genes that decode these most highly used codons. We identify a positive correlation between these parameters (*ρ* = 0.406, *p* = 0.0016, *n* = 61). Dos Reis et al. show that the Relative Adaptiveness to the tRNA pool (w) which takes in account that codons can be recognized by anticodons with perfect or imperfect match (wobble codon-anticodon recognition rules) with different affinities is a better parameter to measure the adaptation of a codon to their decoding tRNAs than the absolute number of tRNA [24]. When the frequency of codon usage was correlated with the Relative Adaptiveness to the tRNA pool (w), an increased correlation was observed (*ρ* = 0.459, *p* = 2.2 × 10^−4^, *n* = 61) (Figure 2).

Synonymous codons are not used equally in an organism. As one tRNA can decode several synonymous codons with different affinities, the expansion of some tRNA families in *C. subvermispora* may be related to the preferential use of certain synonymous codons in coding regions of *C. subvermispora*. To assess this hypothesis, we correlated RSCU values with Relative Adaptiveness to the tRNA pool. Non-statistical correlation was observed, in part because w values are normalized with respect to the tRNA with the highest number of genes able to decode it (*W*_i_/*W*_max_) and not with respect to the pool of tRNAs that decode the complete set of synonymous codons. When W values were normalized with respect to the total amount of tRNAs that decode a set of synonymous codons, a strong correlation with the RSCU values (*ρ* = 0.628, *p* = 0, *n* = 61) was observed. This suggests that among synonymous codons, those highly represented in the *C. subvermispora* genome tend to be decoded by those tRNAs with a high number of gene copies.

### 3.4. Relationship between Gene Expression Level, Codon Bias, and Translational Efficiency in C. subvermispora

The bias in codon usage and adaptation to the tRNA pool modulates translational efficiency. Thus, highly expressed genes tend to use codons that are over-represented in the genome, which in turn present greater availability of tRNAs [24]. To determine whether this relationship exists in *C. subvermispora*, expression levels of *C. subvermispora* genes were correlated with their adaptation values to the tRNA pool and to codon bias. Adaptation to the tRNA pool was evaluated using two indexes: (i) tAI, which measures adaptation of the tRNA pool compared to the relative amount of each tRNA gene, and (ii) AAtAI, which evaluates whether a gene preferentially uses the most abundant tRNA charging a particular amino acid. Codon bias was analyzed by calculation of CAI (Codon Adaptation Index), and CPB (Codon Pair-Bias), to assess if gene expression is correlated with bias in usage of codons or of codon pairs. Codon bias also was evaluated using Nc value to determine if the transcription level is associated with a decrease in the diversity of codons used. We analyzed expression levels determined by RNAseq published by Hori et al. 2014 which was obtained from *C. subvermispora* grown on Ball-Milled Aspen medium [17]. CAI, tAI, AAtAI, and CPB showed high degrees of correlation among them (Table S4), however, these indicators exhibit very low correlation coefficients with expression levels reported by Hori. The high correlation between the different indicators of codon bias and translational efficiency indicates that the frequency with which codons are used in *C. subvermispora* is related to the abundance in the genomes of the tRNAs that decode these codons. 

### 3.5. Transcriptional Response to Growth on Ball-Milled Aspen (BMA), Codon Bias, and Translational Efficiency

**Figure 3:**
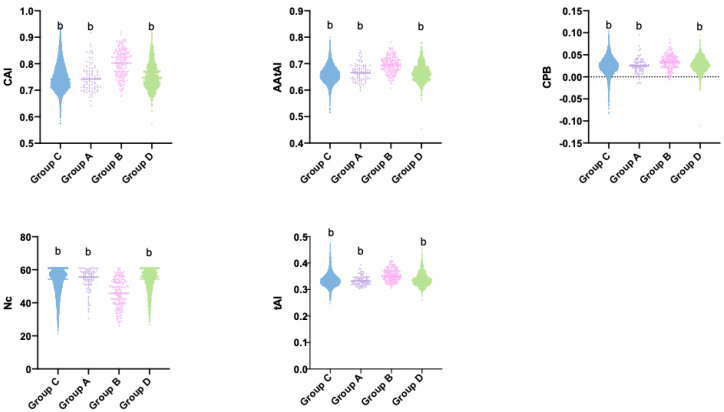
Codon bias and adaptation to tRNA pool of genes induced by Ball-Milled Aspen (BMA). The figure shows the Codon Adaptation Index (CAI), AAtAI, codon pair bias (CPB), effective Number of Codons (Nc), and tAI values of *C. subvermispora* genes. The genes were classified according their change in expression in BMA vs. glucose as carbon source in salt medium, in repressed (*p* < 0.05) at least twofold (Group A), induced (*p* < 0.05) at least twofold (Group B), without statistical changes (*p* > 0.05) (Group C), and genes with slightly (lower than two fold) but significant changes in expression (Group D). Statistical differences between groups were assessed using Mann-Whitney test. b corresponds a *p* < 0.001 in a comparison with Group B.

Growth of *C. subvermispora* in natural environments is dependent on wood. In 2012, Fernandez-Fueyo et al. [8] reported microarray experiments that compared gene expression of *C. subvermispora* grown on glucose and on Ball-Milled Aspen (BMA) as carbon sources. Saline media with BMA has been used as a laboratory medium that mimics growth on wood to analyze expression of genes that are transcriptionally regulated by growth on wood. To analyze if genes regulated by conditions that mimic growth on wood, such as BMA have a different adaptation to the tRNA pool or codon bias, we used the microarray data published by Fernandez-Fueyo [8] and defined four groups of genes: group A corresponds to genes where expression was reduced at least 2 times with a *p*-value lower than 0.05. Group B includes all genes which showed increased expression of at least 2 times with a *p*-value lower than 0.05. Group C corresponds to all genes with non-significant differences (*p* > 0.05) and group D contains all genes with low changes in expression (<2 fold) that are statistically significant. (*p* < 0.05). Our results show that group B has lower values of Nc and higher values of CAI, tAI, AAtAI, and CPB. Groups A, C, and D show non-significant differences among them. This implies that genes induced by wood preferentially use a reduced set of codons that are better adapted to the tRNA pool present in *C. subvermispora* (Figure 3).

When we correlated the CAI, tAI, AAtAI, Nc, and CPB indexes with the ratio between expression in BMA and glucose culture medium, a statistically significant correlation was observed. Positive correlations were found with almost all indicators used (CAI, tAI, AAtAI, and CPB), the exception was Nc that showed a negative correlation. The higher correlation was identified in CAI and tAI indexes, and in genes that showed significant differences for expression in BMA saline medium compared to expression in glucose-supplemented saline medium (Table 2). This increase in correlation coefficients can be explained if growth on lignocellulose exerts pressure on codon usage of genes involved in the metabolization of this carbon source, thereby selecting those codons that increase the translational efficiency of these genes. 

Interestingly, when genes from Group B were sorted according to their codon usage adaptation values or to the tRNA pool, we found that genes coding for ribosomal proteins presented the highest CPB, tAI, and AAtAI values (Table S5). An increase in the expression of ribosomal proteins may lead to improved ribosome biogenesis, which in turn increases the overall protein biosynthetic capacity, as observed in yeast growing in rich media [37,38]. Thus, exposure of *C. subvermispora* to wood or lignocellulose might lead to an increase of the overall translation rate enhancing the synthesis of proteins related to lignocellulose metabolisms. 

### 3.6. Translational Efficiency and Codon Bias in Lignocellulolytic Genes

Genome analysis of *C. subvermispora* indicates that this organism presents an expansion of the number of genes directly related to the mineralization and hydrolysis of lignocellulose. The genome contains 16 annotated genes of ligninolytic peroxidases (13 Manganese peroxidase, one Versatile peroxidase, one lignin peroxidase and one generic peroxidase [9]), seven genes coding for laccases, and 14 genes coding for proteins containing a cellulose-binding domain. Moreover, it also shows an expansion of the auxiliary enzymes required for lignin degradation with four genes for cellobiose dehydrogenases (CDH), five genes for Δ-12 dehydrogenases, four genes for Δ-9 dehydrogenases, five genes for Aryl-alcohol oxidase, four genes for Methanol oxidases, two genes for Aryl alcohol dehydrogenases, three genes for copper radical oxidases, and 14 for glucose methanol choline oxidoreductase [7,8]. Genes belonging to the same family exhibit differential expression, which might reflect that they serve slightly different functions in the mineralization/hydrolysis process of lignocellulose [8]. To determine whether genes of the same family present a similar bias of codon usage and of codon adaptation or adaptation to the tRNA pool, we compared the values of Nc, tAI, AAtAI, CAI, and CPB of these genes. These values were also normalized with respect to the mean and standard deviation of the values obtained for all genes encoded in *C. subvermispora* (*Z*-values) [39]. This normalization was applied to identify if and how values for lignocellulolytic genes differ with respect to these same values in genes not directly related to the lignocellulose degradation processes (Table 3).

Genes encoding manganese peroxidase generally show above-average adaptation values to the tRNA pool and codon usage (*Z*-value > 0). The manganese peroxidase gene with the highest level of expression (transcript ID: 50297) proved to be the most adapted to the tRNA pool, with tAI values that are more than two standard deviations from the mean tAI values of other *C. subvermispora* genes (*Z*-tAI > 2). We also observed that the most highly induced manganese peroxidase gene in BMA medium (transcript ID:129418) also shows a high adaptation value to the tRNA pool (*Z*-tAI = 1.619). Genes encoding laccases showed a similar trend, as the single gene that significantly changed its expression levels after growth in BMA medium (transcript ID: 130783) showed an above average value of tAI (*Z*-tAI = 0.461). 

Among genes encoding for proteins with cellulose binding domains, which includes cellulases and other enzyme that bind and hydrolyzes polymers related to cellulose, the gene encoding for cellulase GH7-CBM1 (transcript ID: 148588) showed the highest adaptation value to the tRNA pool (*Z*-tAI = 1.756) and high expression level under growth with glucose as the sole carbon source. This gene also increased its expression in BMA medium. Additionally, several genes coding for cellulases GH7-CBM1 and GH5-CBM1 showed Nc values below 40, indicating a strong bias in the use of synonymous codons (Table 3). Cellulases from the GH12 family also showed high CAI values. However, in this group of genes no association between expression levels or adaptation to the tRNA pool or any other index used in this work was found.

Genes for CDH show little bias in codon usage (Nc ~ 50). Only the CDH gene which is induced by BMA (transcript ID: 84792) shows an Nc value of 48 and slightly higher than mean (Z > 0) adaptation values to the tRNA pool (Table 3). The Δ12-dehydrogenase genes showed a weak codon bias, with the exception of gene with transcript ID: 124050; this particular gene showed a Nc value of 38 and an above average adaptation to the tRNA pool. This gene also showed strong expression in glucose-containing medium, but its expression was not modified in BMA medium. The opposite behavior was observed in the 9-dehydrogenase genes, where the gene with the greatest induction in BMA (transcript ID: 129048) showed a lower Nc value (41.46), suggesting a strong bias in codon usage (Table 3). Regarding auxiliary genes encoding for Aryl-alcohols oxidase, Methanol oxidase, Aryl-alcohol dehydrogenase and copper radical oxidases, those induced by BMA show CAI and tAI values higher than the average (*Z*-values > 1). An exception were the genes encoding for Methanol oxidase (transcript ID 151964) and Aryl-alcohol dehydrogenase (transcript ID: 126785) which were not induced by BMA but shown *Z*-values > 1.5 (Table S6).

When genes related to mineralization and digestion of lignocellulose were arranged according to their adaptation values to codon usage or the tRNA pool, genes with greater adaptation values encoded ligninolytic peroxidases, and proteins with cellulose-binding domains. Interestingly, manganese peroxidases are more adapted to the tRNA pool, while proteins with cellulose-binding domains showed higher adaptation to codon usage.

High CAI values with tAI values lower than the optimal should indicate the use of frequent codons with low availability of tRNAs to decode them. A reduction of the speed of translation has been associated with the use of rare codons or codons decoded by tRNAs with low availability. This reduction of speed allows the proper folding of the nascent protein. Proteins containing cellulose-binding domains show a complex structure, where a proper folding of at least two domains should require some reduction in the speed of translation [40,41]. Ribosome profiling experiments could help to define if these proteins require coordination between ribosome translocation and protein folding.

## 4. Discussion

The development of massive sequencing technologies, bioinformatics sequence analysis and synthetic biology have established that synonymous mutations, far from being silent, play an important role in the fine-tuning of protein synthesis efficiency and the role of different functional forms [42,43]. The first clues or hints of the importance of synonymous mutations for translational efficiency arose from the identification of bias in the use of synonymous codons that were detected in highly expressed ribosomal proteins from *E. coli*, *Bacillus subtilis*, and *S. cerevisiae* [22,44,45]. In viruses, genes coding for highly required proteins, such as those of the virus capsid or nucleoprotein, show higher adaptation values to codon usage of the host than other viral genes [39,46]. The generation of synthetic genes has revealed that the amount of synthesized protein can vary over thousand fold solely by changing the composition of synonymous codons [47]. Synthetic viruses, constructed from highly virulent viruses, show reduced replication and very low host mortality when their codon usage is changed to codon combinations present in low frequency in the host genome [48]. Further, the arrangement of codons within a gene is not random and is related to the proper folding of the nascent proteins and also to the proper recycling of ribosomes [31]. For example, the low adaptation level of the FRQ gene of *Neurospora crassa* to codon usage is essential to maintain the rhythm of the circadian clock [49]. Continuously increasing evidence supports the fact that bias in the use of synonymous codons plays an active role in the fine-tuning of protein production. The relationship between the use of synonymous codons and translation efficiency lies in the abundance of cognate tRNAs. Highly used codons tend to have a greater number of tRNAs that recognize them [50]. In this work, we have identified 192 genes encoding tRNAs in the white-rot basidiomycete *C. subvermispora*, a number that is similar to the number of tRNA genes present in other fungi such as *Aspergillus fumigatus* (178 genes) and *Schizosaccharomyces pombe* (186 genes) (http://gtrnadb.ucsc.edu/). Moreover, *C. subvermispora* tRNA genes present the interesting feature that each type of tRNA gene (i. e. that loads the same amino acid), groups in a different clade, indicating that the current pool of tRNAs present in the *C. subvermispora* monokaryotic strain B has arisen from gene duplication processes, and probably not from horizontal transfer or recombination. This result is consistent with the absence of a known *C. subvermispora* sexual stage [12]. Interestingly, we identified an expansion of the tRNA genes coding for amino acids glycine and glutamine. The high similarity of their sequences indicates that this expansion may have occurred recently. The expansion of this set of tRNA genes could be the result of the presence of some unidentified SINE elements, which are scored as tRNAs by the tRNAscan program [51]. Another alternative is that this expansion is guided by the need to synthesize large quantities of glycine- and glutamic acid-rich proteins, requiring an enrichment of this set of tRNA genes. This strategy is similar to that used by bacteriophages to increase their rate of protein synthesis in a new host, that involves carrying tRNA genes for those codons that are most frequent in bacteriophage genes [52]. Recently it was discovered that HIV also uses a similar strategy, specifically packaging a pool of tRNAs whose codons are poorly represented in the human genome [53]. Interestingly, the addition of glutamic acid to the culture medium increases the production of cellulases by the brown rot fungus *Fomitopsis* sp. RCK2010 [54], and increases the production of manganese peroxidases and laccases in some white rot fungi [55]. On the other hand, the amino acid glycine is a precursor of heme biosynthesis [56], an important cofactor present in manganese peroxidases and cytochromes, genes that are highly abundant in *C. subvermispora*.

We also found a strong bias in codon usage, codon pairs, and adaptation to the tRNA pool of *C. subvermispora* genes involved in lignocellulose degradation. Similar bias has been described in lignin peroxidase from *Phaenorochaete chrysosporium* [57]. Since a bioinformatic identification of these genes can only be applied to those directly involved in the mineralization/digestion of lignocellulose, such as manganese peroxidase type enzymes, laccases, and cellulases, we devised a functional definition, whereby genes involved in lignocellulose degradation are those induced in the presence of BMA, a model substrate of wood (Group B). Using this functional definition, we were able to detect biases for all evaluated parameters. In turn, the lack of a strong correlation between gene expression levels and adaptation to the tRNA pool or codon bias of *C. subvermispora* grown in a medium containing glucose or BMA, is consistent with this postulate. A similar correlation between gene function, levels of transcriptional induction, and adaption to the translational machinery is expected when *C. subvermispora* grown on wood or other media that resemble its natural substrates. Future experiments need to be addressed to test this hypothesis. Studies of phylogenetic reconstruction indicate that the lignocellulose-degrading machinery may have arisen during the Paleozoic [58]. It is reasonable to pose that efficient use of this carbon source requires a metabolic adaptation that involves the whole organism, in addition to the emergence of new types of enzymes. Therefore, it is expected that genes encoding for enzymes that are part of the general metabolism of *C. subvermispora* also respond to exposure of lignocellulose, as observed in microarray experiments reported by Fernandez Fueyo et al. 2012 [8] were 293 genes modify significantly its expression at least 2 fold (205 from Group B and 88 from Group A). Among them, we can find genes related to the Krebs cycle, cell division, and translation (Table S5). 

Expansion of cellulase, laccase, and manganese peroxidase gene families in the *C. subvermispora* genome permitted that each of these genes developed differential expression; however, the precise contribution of each of these genes in the digestion and mineralization of lignocellulose is not clear. In bacteria, an increase of the copy number of a particular gene is a strategy for increasing its expression. This genomic adaptation explains the expansion of genes directly related to the digestion and mineralization of lignocellulose. However, the complex expression pattern of these genes suggests that this gene expansion process is more intricate than simply a response to the need of producing more enzymes. The high bias in codon usage, adaptation of the tRNA pool, and codon diversity, in some genes directly related to the digestion and mineralization processes of lignocellulose together with the increase in expression of ribosomal proteins in BMA, suggest that *C. subvermispora* also uses increased translational efficiency as an additional strategy to increase the production these specific set of proteins. Thus, the increase in copy number may be linked to the generation of a diverse array of enzymes that can process a wide range of substrates. In support of this hypothesis, it has been shown that *C. subvermipora* manganese peroxidases present different kinetic parameters and substrate specificities [8,9].

## 5. Conclusions

Our results suggest that lignocellulose degradation by *C. subvermispora* has modified the genome structure of this fungus, changing the bias in codon usage, the tRNA gene pool, and codon diversity in genes that are induced in the presence of wood substrates, in order to optimize the production of these proteins. This strategy may be particularly useful in slow-growing organisms, such as *C. subvermispora* that cannot increase the production of enzymes by increasing cell mass. To our knowledge, this study is the first example to show metabolic adaptation to a particular ecological niche by modification of the genetic structure of an organism in favor of a selective increase of the translational efficiency of genes involved in metabolizing specific substrates that determine its adaptation to a particular environment.

## Figures and Tables

**Table 1:** Statistics of tRNAs present in the *Ceriporiopsis subvermispora* genome.

tRNA Type	Number of tRNAs	Anticodon and Frequency *	Scaffolds	Number of tRNAs with Introns
Ala	14	AGC^7^, CGC^5^, TGC^2^	3, 5, 7, 12, 14, 18, 19	13
Arg	16	ACG^8^, CCG^2^, CCT^2^, TCG^3^, TCT^1^	2, 3, 5, 9, 14, 19, 31, 39	15
Asn	5	GTT^5^	4, 5, 7, 13, 14	5
Asp	10	GTC^10^	1, 6, 8, 12, 16, 19, 28, 41	7
Cys	3	GCA^3^	10, 13, 20	1
Gln	8	CTG^6^, TTG^2^	1, 2, 6, 17, 18, 28	8
Glu	15	CTC^12^, TTC^3^	3, 8, 9, 19, 28	5
Gly	17	CCC^2^, GCC^12^, TCC^3^	1, 2, 5, 7, 9, 10, 13, 20	1
His	5	GTG^5^	2, 6, 7, 12	5
Ile	9	AAT^8^, TAT^1^	4, 5, 21, 33	9
Leu	15	AAG^7^, CAA^2^, CAG^4^, TAA^1^, TAG^1^	1, 2, 4, 5, 6, 7, 9, 13, 17, 25, 27, 30	14
Lys	11	CTT^10^, TTT^1^	1, 3	11
Met	8	CAT^8^	1, 2, 3, 9, 10, 11, 12, 23	7
Phe	5	GAA^5^	1, 5, 9, 10	4
Pro	10	AGG^5^, CGG^4^, TGG^1^	4, 6, 7, 14, 18, 22, 35	10
Ser	13	ACT^1^, AGA^4^, CGA^3^, GCT^3^, TGA^2^	1, 3, 4, 5, 6, 9, 15, 18	8
Thr	9	AGT^6^, CGT^2^, TGT^1^	1, 3, 4, 8, 9, 10, 22	6
Trp	3	CCA^3^	19, 20, 30	3
Tyr	4	GTA^4^	2, 3, 8, 30	4
Val	11	TAC^1^, CAC^2^, AAC^6^, CAC^1^, AAC^1^	5, 6, 12, 14, 15, 28, 32	2

* Frequency is indicated as superscript together the anticodon

**Table 2:** Correlation coefficient between gene expression, codon bias, and translational efficiency.

	BMA (*ρ*)	Glu (*ρ*)	BMA/Glu (*ρ*)	BMA/Glu (*ρ*) ^a^	BMA/Glu (*ρ*) ^b^	BMA/Glu (*ρ*) ^c^
CAI	−4.95 × 10^−3^	−8.04 × 10^−2^ ***	3.39 × 10^−1^ ***	4.20 × 10^−1^ **	4.66 × 10^−1^ ***	3.09 × 10^−1^ ***
CPB	6.09 × 10^−2^ ***	6.59 × 10^−3^ ***	2.61 × 10^−1^ ***	NA	3.26 × 10^−1^ ***	2.47 × 10^−1^ ***
Nc	1.81 × 10^−2^ *	8.17 × 10^−2^ ***	−2.63 × 10^−1^ ***	−4.51 × 10^−1^ ***	−4.42 × 10^−1^ ***	−2.21 × 10^−1^ ***
tAI	3.48 × 10^−2^ ***	−3.52 × 10^−2^ ***	3.17 × 10^−1^ ***	2.84 × 10^−1^ *	4.36 × 10^−1^ ***	2.82 × 10^−1^ ***
AAtAI	6.54 × 10^−2^ ***	8.34 × 10^−4^	2.67 × 10^−1^ ***	4.75 × 10^−1^ ***	4.17 × 10^−1^ ***	2.27 × 10^−1^ ***

* *p* < 0.05 ** *p* < 0.01 *** *p* < 0.001. ^a^ Genes with *p*-value lower than 0.001, *n* = 52. ^b^ Genes with *p*-value between 0.001 and 0.05, *n* = 1572. ^c^ Genes with *p*-value higher than 0.05, *n* = 10,471. Glu = Glucose.

**Table 3:** Adaptation to codon usage, tRNA pool, and codon bias of genes from *C. subvermispora* involved in mineralization and digestion of lignocellulose. Bold letters indicate the five highest values.

Ligno-Cellulolytic Function	Transcript ID	CAI	AAtAI	tAI	CPB	Nc	Z-CAI	Z-AAtAI	Z-CPB	Z-tAI	Putative Function	Microarray Signal (log2)			
												Glucose	BMA	BMA/Glu	*p*-Value
Fungal lignin peroxidase	49863	0.814	0.720	0.371	0.028	40.95	1.236	1.848	0.136	1.513	Peroxidase, MnP	9.58	11.15	2.970	0.08320
	50297	0.822	0.735	**0.384**	**0.055**	41.46	1.394	2.321	**1.565**	**2.111**	Peroxidase, MnP	13.13	11.88	0.421	0.19000
	50686	0.823	0.728	**0.379**	**0.051**	41.50	1.414	2.100	**1.387**	**1.901**	Peroxidase, MnP	9.35	9.42	1.048	0.45600
	106380	0.815	0.716	0.371	0.039	38.67	1.255	1.722	0.705	1.536	Peroxidase, MnP	8.93	8.93	1.000	0.99900
	111364	0.770	0.689	0.357	0.015	50.57	0.364	0.872	−0.611	0.929	Peroxidase, VP-like	9.18	9.11	0.957	0.52800
	117521	0.819	0.722	0.367	0.042	40.88	1.335	1.911	0.876	1.340	Peroxidase, MnP	9.57	9.49	0.945	0.30600
	124144	0.696	0.653	0.321	0.031	61.00	−1.101	−0.261	0.284	−0.660	Peroxidase, generic	11.30	11.21	0.942	0.29300
	126018	0.828	0.721	0.366	0.030	40.55	1.513	1.880	0.230	1.314	Peroxidase, MnP	9.69	9.50	0.874	0.10000
	126058	0.788	0.673	0.344	0.026	44.68	0.721	0.368	−0.015	0.340	Peroxidase, MnP	9.94	9.44	0.707	***0.00595***
	128590	0.824	0.724	0.367	**0.052**	41.12	1.434	1.974	**1.409**	1.352	Peroxidase, MnP	9.78	9.52	0.837	***0.01960***
	129418	0.767	0.709	**0.373**	0.044	49.06	0.305	1.502	1.011	**1.619**	Peroxidase, MnP	10.22	12.92	6.508	***0.00895***
	130659	0.781	0.721	0.368	0.022	50.53	0.582	1.880	−0.220	1.396	Peroxidase, LiP-like	10.88	10.30	0.673	***0.03900***
	136058	0.770	0.688	0.352	0.029	50.91	0.364	0.841	0.167	0.683	Peroxidase, MnP	10.16	10.76	1.519	0.16700
	151947	0.831	0.729	**0.373**	**0.052**	40.42	1.572	2.132	**1.410**	**1.600**	Peroxidase, MnP	9.00	8.93	0.955	0.24200
	155372	0.797	0.711	0.369	0.040	42.88	0.899	1.565	0.765	1.443	Peroxidase, MnP	8.86	8.84	0.988	0.74100
	169968	0.816	0.718	0.369	0.032	42.96	1.275	1.785	0.359	1.434	Peroxidase, MnP	10.27	10.16	0.930	0.44200
Laccase	84170	0.741	0.664	0.325	0.032	58.64	−0.210	0.085	0.338	−0.517	laccase	10.45	10.34	0.931	0.17000
	88089	0.841	0.684	0.336	0.039	41.00	1.770	0.715	0.695	−0.016	Laccase	9.38	9.29	0.939	0.17800
	120834	0.737	0.669	0.330	0.011	57.80	−0.289	0.242	−0.825	−0.271	Laccase	10.74	10.66	0.945	0.50800
	127045	0.752	0.681	0.339	0.024	54.32	0.008	0.620	−0.103	0.112	Laccase	10.54	10.02	0.694	***0.00260***
	127050	0.721	0.667	0.334	0.015	60.26	−0.606	0.179	−0.581	−0.098	Laccase	11.35	11.19	0.895	0.54300
	130783	0.791	0.705	0.347	0.037	43.13	0.780	1.376	0.605	0.461	Laccase	11.02	13.77	6.766	***0.00426***
	149668	0.775	0.679	0.334	0.019	51.25	0.463	0.557	−0.397	−0.120	Laccase	9.91	9.81	0.931	0.35300
Cellulose Binding Protein	59733	0.812	0.704	0.346	0.026	46.11	1.196	1.344	−0.018	0.414	GH10-CBM1	9.24	13.69	21.783	***0.00004***
	66688	0.824	0.716	0.347	0.025	47.02	1.434	1.722	−0.048	0.488	GH61-CBM1	9.62	14.87	37.901	***0.00002***
	67561	0.853	0.730	0.366	0.020	39.43	2.008	2.163	−0.336	1.313	GH10-CBM1	10.30	13.71	10.642	***0.00002***
	68569	0.837	0.735	0.345	0.024	43.59	1.691	2.321	−0.095	0.383	CE1-CBM1	9.80	13.10	9.858	***0.00023***
	79557	0.802	0.715	0.345	0.022	42.15	0.998	1.691	−0.231	0.373	GH5-CBM1	10.24	14.04	13.874	***0.00001***
	87580	0.783	0.693	0.321	0.010	49.31	0.622	0.998	−0.884	−0.692	CE16-CBM1	10.94	14.46	11.540	***0.00004***
	89533	0.838	**0.736**	0.353	**0.052**	42.06	1.711	**2.352**	**1.426**	0.719	GH61-CBM1	10.35	13.56	9.306	***0.00002***
	89534	**0.870**	0.731	0.352	0.044	**37.58**	**2.345**	2.195	0.980	0.693	GH61-CBM1	9.42	10.13	1.637	***0.02300***
	101925	0.845	0.723	0.359	0.033	38.22	1.850	1.943	0.394	1.020	GH7-CBM1	8.84	8.96	1.086	0.09060
	106777	0.804	0.712	0.348	0.035	49.87	1.038	1.596	0.511	0.500	GH5- CBM1	9.54	14.35	28.050	***0.00001***
	109840	**0.857**	**0.741**	0.370	0.039	**38.16**	**2.087**	**2.509**	0.733	1.471	GH10- CBM1	9.52	11.85	5.009	***0.00053***
	129028	0.852	0.733	0.363	0.042	39.91	1.988	2.258	0.849	1.181	GH5- CBM1	9.81	10.79	1.985	***0.01830***
	133809	**0.865**	0.713	0.338	0.040	**37.08**	**2.246**	1.628	0.779	0.091	GH11-CBM1	10.86	11.42	1.466	***0.02090***
	148588	**0.856**	**0.742**	**0.376**	0.008	**34.45**	**2.067**	**2.541**	−0.967	**1.756**	GH7-CBM1	11.02	12.62	3.015	***0.00018***
CDH	84792	0.803	0.688	0.338	0.023	48.09	1.018	0.841	−0.137	0.071	CDH	9.29	13.76	22.241	***0.00002***
	87110	0.769	0.679	0.332	0.024	50.71	0.345	0.557	−0.111	−0.189		11.31	11.04	0.827	0.20200
	125610	0.762	0.665	0.318	0.044	53.34	0.206	0.116	0.981	−0.814	cir1 CBM1	10.17	10.40	1.170	0.23300
	147544	0.712	0.665	0.318	0.025	56.98	−0.784	0.116	−0.060	−0.798		11.24	11.02	0.860	0.19900
Delta 12 Dehidrogenase	58880	0.727	0.670	0.317	0.008	59.40	−0.487	0.274	−0.973	−0.853	Δ-12 FAD	10.36	10.29	0.956	0.72900
	121074	0.731	0.644	0.313	0.022	55.49	−0.408	−0.545	−0.219	−1.033	Δ-12 FAD	10.58	10.23	0.783	***0.01490***
	124050	**0.856**	**0.736**	0.368	0.042	**38.22**	**2.067**	**2.352**	0.875	1.416	Δ-12 FAD. Cs-fad2	12.74	12.66	0.941	0.65300
	136101	0.714	0.649	0.310	0.007	56.78	−0.745	−0.387	−1.016	−1.178	Δ-12 FAD	11.01	12.54	2.895	***0.00022***
	167690	0.736	0.670	0.325	0.013	58.45	−0.309	0.274	−0.706	−0.484	Δ-12 FAD	10.67	10.11	0.678	***0.00870***
Delta 9 Dehidrogenase	87875	0.810	0.694	0.343	0.035	44.18	1.156	1.030	0.475	0.313	Δ-9 FAD, Cs-ole1	8.93	8.94	1.006	0.88000
	129045	0.728	0.679	0.335	0.045	56.92	−0.467	0.557	1.017	−0.075	Δ-9 FAD, Cs-ole1	8.95	8.91	0.974	0.52700
	129048	0.848	**0.740**	0.366	0.050	41.46	1.909	**2.478**	1.291	1.292	Δ-9 FAD, Cs-ole1	11.78	12.35	1.478	***0.02980***
	133675	0.760	0.669	0.330	0.024	54.62	0.166	0.242	−0.113	−0.266	Δ-9 FAD, Cs-ole1	9.64	9.51	0.917	0.17900

## Supplementary Materials

The following are available online at https://www.mdpi.com/2073-4425/11/10/1227/s1, Table S1: CPS values of pair-codon present in C. subvermispora, Table S2: tRNA encoded in the Draft genome of C. subvermispora, Table S3: tRNA repeated in C. subvermispora, Table S4: Correlation coefficient among indicators. Table S5: Values of CAI, tAI, AAtAI, Nc, and CPB in C. subvermipora genes expressed in Salt medium with BMA or Glucose. Table S6: Codon bias and translation efficiency of genes encoding for enzymes auxiliary in the lignocellulose degradation.

## References

[1] Martínez A.T., Speranza M., Ruiz-Dueñas F.J., Ferreira P., Camarero S., Guillén F., Martínez M.J., Gutiérrez A., del Río J.C. (2005). Biodegradation of lignocellulosics: Microbial, chemical, and enzymatic aspects of the fungal attack of lignin. Int. Microbiol..

[2] Kersten P., Cullen D. (2007). Extracellular oxidative systems of the lignin-degrading Basidiomycete Phanerochaete chrysosporium. Fungal Genet. Biol..

[3] Wan C., Li Y. (2012). Fungal pretreatment of lignocellulosic biomass. Biotechnol. Adv..

[4] Baldrian P., Valásková V. (2008). Degradation of cellulose by basidiomycetous fungi. FEMS Microbiol. Rev..

[5] Rüttimann-Johnson C., Salas L., Vicuña R., Kirk T.K. (1993). Extracellular Enzyme Production and Synthetic Lignin Mineralization by Ceriporiopsis subvermispora. Appl. Environ. Microbiol..

[6] Lobos S., Larraín J., Salas L., Cullen D., Vicuña R. (1994). Isoenzymes of manganese-dependent peroxidase and laccase produced by the lignin-degrading basidiomycete Ceriporiopsis subvermispora. Microbiology.

[7] Enoki M., Watanabe T., Nakagame S., Koller K., Messner K., Honda Y., Kuwahara M. (1999). Extracellular lipid peroxidation of selective white-rot fungus, Ceriporiopsis subvermispora. FEMS Microbiol. Lett..

[8] Fernandez-Fueyo E., Ruiz-Dueñas F.J., Ferreira P., Floudas D., Hibbett D.S., Canessa P., Larrondo L.F., James T.Y., Seelenfreund D., Lobos S. (2012). Comparative genomics of Ceriporiopsis subvermispora and Phanerochaete chrysosporium provide insight into selective ligninolysis. Proc. Natl. Acad. Sci. USA.

[9] Fernández-Fueyo E., Ruiz-Dueñas F.J., Miki Y., Martínez M.J., Hammel K.E., Martínez A.T. (2012). Lignin-degrading peroxidases from genome of selective ligninolytic fungus Ceriporiopsis subvermispora. J. Biol. Chem..

[10] Salas C., Lobos S., Larraín J., Salas L., Cullen D., Vicuña R. (1995). Properties of laccase isoenzymes produced by the basidiomycete Ceriporiopsis subvermispora. Biotechnol. Appl. Biochem..

[11] Tello M., Corsini G., Larrondo L.F., Salas L., Lobos S., Vicuña R. (2000). Characterization of three new manganese peroxidase genes from the ligninolytic basidiomycete Ceriporiopsis subvermispora. Biochim. Biophys. Acta.

[12] Tello M., Seelenfreund D., Lobos S., Gaskell J., Cullen D., Vicuña R. (2001). Isolation and characterization of homokaryotic strains from the ligninolytic basidiomycete Ceriporiopsis subvermispora. FEMS Microbiol. Lett..

[13] Manubens A., Canessa P., Folch C., Avila M., Salas L., Vicuña R. (2007). Manganese affects the production of laccase in the basidiomycete Ceriporiopsis subvermispora. FEMS Microbiol. Lett..

[14] Gutiérrez M., Rojas L.A., Mancilla-Villalobos R., Seelenfreund D., Vicuña R., Lobos S. (2008). Analysis of manganese-regulated gene expression in the ligninolytic basidiomycete Ceriporiopsis subvermispora. Curr. Genet..

[15] Alvarez J.M., Canessa P., Mancilla R.A., Polanco R., Santibáñez P.A., Vicuña R. (2009). Expression of genes encoding laccase and manganese-dependent peroxidase in the fungus Ceriporiopsis subvermispora is mediated by an ACE1-like copper-fist transcription factor. Fungal Genet. Biol..

[16] Mancilla R.A., Canessa P., Manubens A., Vicuña R. (2010). Effect of manganese on the secretion of manganese-peroxidase by the basidiomycete Ceriporiopsis subvermispora. Fungal Genet. Biol..

[17] Hori C., Gaskell J., Igarashi K., Kersten P., Mozuch M., Samejima M., Cullen D. (2014). Temporal alterations in the secretome of the selective ligninolytic fungus Ceriporiopsis subvermispora during growth on aspen wood reveal this organism’s strategy for degrading lignocellulose. Appl. Environ. Microbiol..

[18] Manubens A., Avila M., Canessa P., Vicuña R. (2003). Differential regulation of genes encoding manganese peroxidase (MnP) in the basidiomycete Ceriporiopsis subvermispora. Curr. Genet..

[19] Sharp P.M., Stenico M., Peden J.F., Lloyd A.T. (1993). Codon usage: Mutational bias, translational selection, or both?. Biochem. Soc. Trans..

[20] Supek F., Skunca N., Repar J., Vlahovicek K., Smuc T. (2010). Translational selection is ubiquitous in prokaryotes. PLoS Genet..

[21] Tuller T., Waldman Y.Y., Kupiec M., Ruppin E. (2010). Translation efficiency is determined by both codon bias and folding energy. Proc. Natl. Acad. Sci. USA.

[22] Sharp P.M., Li W.H. (1987). The codon Adaptation Index--a measure of directional synonymous codon usage bias, and its potential applications. Nucleic Acids Res..

[23] Bulmer M. (1987). Coevolution of codon usage and transfer RNA abundance. Nature.

[24] dos Reis M., Savva R., Wernisch L. (2004). Solving the riddle of codon usage preferences: A test for translational selection. Nucleic Acids Res..

[25] Lowe T.M., Eddy S.R. (1997). tRNAscan-SE: A program for improved detection of transfer RNA genes in genomic sequence. Nucleic Acids Res..

[26] Carver T., Bleasby A. (2003). The design of Jemboss: A graphical user interface to EMBOSS. Bioinformatics.

[27] Tello M., Saavedra J.M., Spencer E. (2013). Analysis of the use of codon pairs in the HE gene of the ISA virus shows a correlation between bias in HPR codon-pair use and mortality rates caused by the virus. Virol. J..

[28] Coleman J.R., Papamichail D., Skiena S., Futcher B., Wimmer E., Mueller S. (2008). Virus attenuation by genome-scale changes in codon pair bias. Science.

[29] Mueller S., Papamichail D., Coleman J.R., Skiena S., Wimmer E. (2006). Reduction of the rate of poliovirus protein synthesis through large-scale codon deoptimization causes attenuation of viral virulence by lowering specific infectivity. J. Virol..

[30] Rice P., Longden I., Bleasby A. (2000). EMBOSS: The European Molecular Biology Open Software Suite. Trends Genet..

[31] Tuller T., Carmi A., Vestsigian K., Navon S., Dorfan Y., Zaborske J., Pan T., Dahan O., Furman I., Pilpel Y. (2010). An evolutionarily conserved mechanism for controlling the efficiency of protein translation. Cell.

[32] Thompson J.D., Higgins D.G., Gibson T.J. (1994). CLUSTAL W: Improving the sensitivity of progressive multiple sequence alignment through sequence weighting, position-specific gap penalties and weight matrix choice. Nucleic Acids Res..

[33] Saitou N., Nei M. (1987). The neighbor-joining method: A new method for reconstructing phylogenetic trees. Mol. Biol. Evol..

[34] Dopazo J. (1994). Estimating errors and confidence intervals for branch lengths in phylogenetic trees by a bootstrap approach. J. Mol. Evol..

[35] Tamura K., Nei M., Kumar S. (2004). Prospects for inferring very large phylogenies by using the neighbor-joining method. Proc. Natl. Acad. Sci. USA.

[36] Tamura K., Peterson D., Peterson N., Stecher G., Nei M., Kumar S. (2011). MEGA5: Molecular evolutionary genetics analysis using maximum likelihood, evolutionary distance, and maximum parsimony methods. Mol. Biol. Evol..

[37] Fingerman I., Nagaraj V., Norris D., Vershon A.K. (2003). Sfp1 plays a key role in yeast ribosome biogenesis. Eukaryot. Cell.

[38] Marion R.M., Regev A., Segal E., Barash Y., Koller D., Friedman N., O’Shea E.K. (2004). Sfp1 is a stress- and nutrient-sensitive regulator of ribosomal protein gene expression. Proc. Natl. Acad. Sci. USA.

[39] Tello M., Vergara F., Spencer E. (2013). Genomic adaptation of the ISA virus to Salmo salar codon usage. Virol. J..

[40] Zhao V., Jacobs W.M., Shakhnovich E.I. (2020). Effect of protein structure on evolution of cotranslational folding. Biophys. J..

[41] Bitran A., Jacobs W.M., Zhai X., Shakhnovich E. (2020). Cotranslational folding allows misfolding-prone proteins to circumvent deep kinetic traps. Proc. Natl. Acad. Sci. USA.

[42] Aragonès L., Guix S., Ribes E., Bosch A., Pintó R.M. (2010). Fine-tuning translation kinetics selection as the driving force of codon usage bias in the hepatitis A virus capsid. PLoS Pathog..

[43] Plotkin J.B., Kudla G. (2011). Synonymous but not the same: The causes and consequences of codon bias. Nat. Rev. Genet..

[44] Sharp P.M., Tuohy T.M., Mosurski K.R. (1986). Codon usage in yeast: Cluster analysis clearly differentiates highly and lowly expressed genes. Nucleic Acids Res..

[45] Sharp P.M., Cowe E., Higgins D.G., Shields D.C., Wolfe K.H., Wright F. (1988). Codon usage patterns in Escherichia coli, Bacillus subtilis, Saccharomyces cerevisiae, Schizosaccharomyces pombe, Drosophila melanogaster and Homo sapiens; a review of the considerable within-species diversity. Nucleic Acids Res..

[46] Bahir I., Fromer M., Prat Y., Linial M. (2009). Viral adaptation to host: A proteome-based analysis of codon usage and amino acid preferences. Mol. Syst. Biol..

[47] Angov E. (2011). Codon usage: Nature’s roadmap to expression and folding of proteins. Biotechnol. J..

[48] Mueller S., Coleman J.R., Papamichail D., Ward C.B., Nimnual A., Futcher B., Skiena S., Wimmer E. (2010). Live attenuated influenza virus vaccines by computer-aided rational design. Nat. Biotechnol..

[49] Zhou M., Guo J., Cha J., Chae M., Chen S., Barral J.M., Sachs M.S., Liu Y. (2013). Non-optimal codon usage affects expression, structure and function of clock protein FRQ. Nature.

[50] Novoa E.M., Pavon-Eternod M., Pan T., de Pouplana L.R. (2012). A role for tRNA modifications in genome structure and codon usage. Cell.

[51] Wenke T., Döbel T., Sörensen T.R., Junghans H., Weisshaar B., Schmidt T. (2011). Targeted identification of short interspersed nuclear element families shows their widespread existence and extreme heterogeneity in plant genomes. Plant Cell.

[52] Enav H., Béjà O., Mandel-Gutfreund Y. (2012). Cyanophage tRNAs may have a role in cross-infectivity of oceanic Prochlorococcus and Synechococcus hosts. ISME J..

[53] van Weringh A., Ragonnet-Cronin M., Pranckeviciene E., Pavon-Eternod M., Kleiman L., Xia X. (2011). HIV-1 modulates the tRNA pool to improve translation efficiency. Mol. Biol. Evol..

[54] Deswal D., Khasa Y.P., Kuhad R.C. (2011). Optimization of cellulase production by a brown rot fungus Fomitopsis sp. RCK2010 under solid state fermentation. Bioresour. Technol..

[55] Levin L., Melignani E., Ramos A.M. (2010). Effect of nitrogen sources and vitamins on ligninolytic enzyme production by some white-rot fungi. Dye decolorization by selected culture filtrates. Bioresour. Technol..

[56] Heinemann I.U., Jahn M., Jahn D. (2008). The biochemistry of heme biosynthesis. Arch. Biochem. Biophys..

[57] Ritch T.G., Gold M.H. (1992). Characterization of a highly expressed lignin peroxidase-encoding gene from the basidiomycete Phanerochaete chrysosporium. Gene.

[58] Floudas D., Binder M., Riley R., Barry K., Blanchette R.A., Henrissat B., Martínez A.T., Otillar R., Spatafora J.W., Yadav J.S. (2012). The Paleozoic origin of enzymatic lignin decomposition reconstructed from 31 fungal genomes. Science.

